# Biohumoral Indicators Influenced by Physical Activity in the Elderly

**DOI:** 10.3390/jcm9041115

**Published:** 2020-04-13

**Authors:** Chiara Fossati, Guglielmo Torre, Paolo Borrione, Arrigo Giombini, Federica Fagnani, Matteo Turchetta, Erika Albo, Maurizio Casasco, Attilio Parisi, Fabio Pigozzi

**Affiliations:** 1 Department of Movement, Human and Health Sciences, University of Rome “Foro Italico”, 00135 Rome, Italy; chiara.fossati@uniroma4.it (C.F.); paolo.borrione@uniroma4.it (P.B.); arrigo.giombini@uniroma4.it (A.G.); federica.fagnani@uniroma4.it (F.F.); attilio.parisi@uniroma4.it (A.P.); fabio.pigozzi@uniroma4.it (F.P.); 2 Department of Orthopaedic and Trauma Surgery, Campus Bio-Medico University of Rome, 00128 Roma, Italy; e.albo@unicampus.it; 3 Department of Orthopaedics, Policlinico Casilino, 00169 Rome, Italy; matteoturchetta1@gmail.com; 4 Italian Federation of Sports Medicine, 00196 Rome, Italy; presidente@fmsi.it

**Keywords:** physical activity, elderly, biomarkers, noncommunicable diseases, hypertension, diabetes

## Abstract

In the scientific landscape, there is a growing interest in defining the role of several biomolecules and humoral indicators of the aging process and in the modifications of these biomarkers induced by physical activity and exercise. The main aim of the present narrative review is to collect the available evidence on the biohumoral indicators that could be modified by physical activity (PA) in the elderly. Online databases including Pubmed, Web of science (Medline), and Scopus were searched for relevant articles published in the last five years in English. Keywords and combination of these used for the search were the following: “biological”, “indicators”, “markers”, “physical”, “activity”, and “elderly”. Thirty-four papers were analyzed for inclusion. Twenty-nine studies were included and divided into four categories: cardiovascular (CV) biomarkers, metabolic biomarkers, inflammatory markers-oxidative stress molecules, and other markers. There are many distinct biomarkers influenced by PA in the elderly, with promising results concerning the metabolic and CV indexes, as a growing number of studies demonstrate the role of PA on improving parameters related to heart function and CV risk like atherogenic lipid profile. Furthermore, it is also a verified hypothesis that PA is able to modify the inflammatory status of the subject by decreasing the levels of pro-inflammatory cytokines, including interleukin-1 (IL-1), interleukin-6 (IL-6), and tumor necrosis factor-alpha (TNF-α). PA seems also to be able to have a direct effect on the immune system. There is a strong evidence of a positive effect of PA on the health of elderly people that could be evidenced and “quantified” by the modifications of the levels of several biohumoral indicators.

## 1. Introduction

Successful aging is one of the main health-related concerns of nowadays, as the public burden related to aging becomes even more consistent, especially in terms of assistance and expense. Physical activity (PA) plays a key role in aging, as many studies have evinced its beneficial effects in primary and secondary prevention of noncommunicable diseases (NCDs) and its influence in the aging process of tissues. Furthermore, PA has a relevant role in mental wellness, in decreasing dementia and stress reactions [1], as well as in increasing the life expectancy. The World Health Organization (WHO) recommends a minimum of 150 min of moderate exercise or 75 min of vigorous training per week for adults, including older adults. Physical inactivity is considered the fourth major risk factor for global mortality [1], leading to approximately 3 million deaths per year [2]. According to actual data from Europe, there is a lack of active lifestyles in daily life, as older adults spend an average of 9.4 h per day in sedentary activities [3] and only 7% practice regular exercise [1]. Age is indeed the most relevant risk factor for inactivity [4], since for older people, it is significantly more challenging to get engaged in exercise when compared to younger subjects. Conceivably, a comprehensible vicious circle develops, since the presence of systemic diseases prevents the subject from being engaged in exercise and the lack of activity yields a worsening of such systemic pathologic conditions. A major role of physical inactivity has been reported in the onset and worsening of cardiovascular (CV) disease, type 2 diabetes mellitus (T2DM), atherosclerosis, neurodegeneration, and cancer. The combination of these diseases as well as the presence of osteoporosis and sarcopenia configures a frailty syndrome, considered the main cause of disability in the elderly [5].

One of the main challenges of modern medicine and public health is efficient monitoring of health conditions of the elderly to better understand the aging process and to prevent the development of such a vicious mechanism. In the scientific landscape, there is a growing interest in defining the role of several biomolecules and humoral indicators [6] of the aging process and in the modifications of these biomarkers induced by physical activity and exercise [7]. However, the role of PA on these modifications is far from being completely elucidated.

The main objective of the present narrative review is to collect the available evidence above the biohumoral indicators that could be modified by PA in the elderly in order to understand by the use of quantitative indicators its role in protecting the health and functional wellbeing of this age group.

## 2. Methods

### 2.1. Criteria for Considering Studies for This Review

Types of studies considered for inclusion in the present review were randomized controlled trials (RCT), prospective cohort studies (PCS), case-control studies (CCS), and letters to the editor. Studies considered should concern biohumoral indicators, biomolecules, and markers that are influenced by PA in elderly.

### 2.2. Search Methods for Identification of Studies

Online databases including Pubmed, Web of science (Medline), and Scopus were searched for relevant articles published in the last five years in English. Keywords and combination of these used for the search were the following: “biological”, “indicators”, “markers”, “physical”, “activity”, and “elderly”. The studies retrieved were firstly screened by title, and then, the whole abstract was examined for the relevant ones.

**Figure 1:**
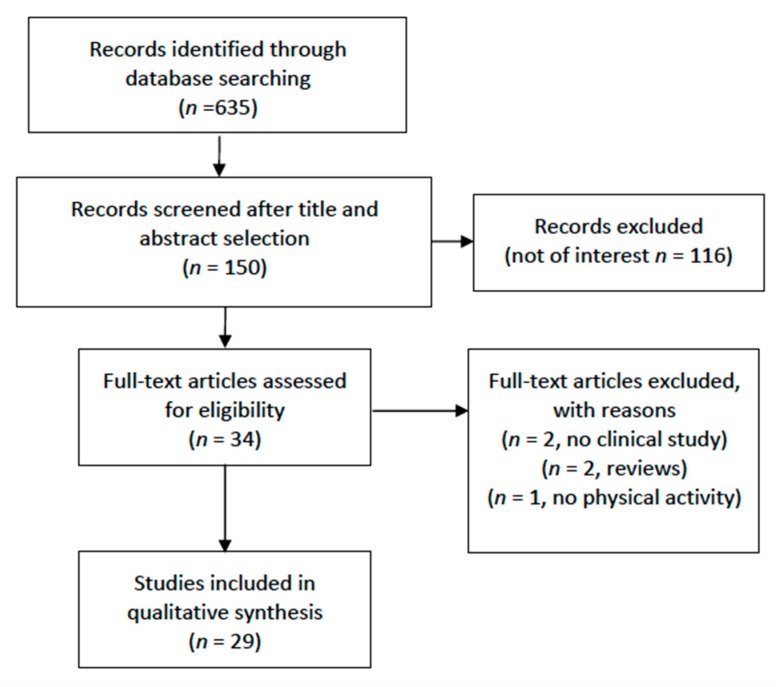
Flow chart of the inclusion process.

After a first selection and exclusion of nonrelevant papers and papers which did not focus on elderly (mean age < 60 years old), the full-text of the potentially eligible articles was retrieved and read for possible inclusion. To enrich the electronic search with further studies, the bibliography of the relevant articles was manually searched to identify potentially eligible papers missed at the electronic search. Thirty-four papers were analyzed for inclusion, 3 of these were then excluded (2 did not concern clinical research, and 1 investigated functional status and not PA). Two more were excluded because they were systematic reviews (Figure 1). Studies included were divided into four categories depending on the type of biohumoral indicators that were investigated: CV biomarkers, metabolic biomarkers, inflammatory markers-oxidative stress molecules, and other markers. The following categories of population were considered by the studies included in the review: community dwelling “healthy” elderly, elderly with pathology/activity limitation, frail elderly, hypertensive patients, diabetic patients, metabolic syndrome patients, and breast cancer patients. After extraction of data concerning the paper, a summary of the results was reported in the text.

## 3. Results

### 3.1. Effect of PA on Inflammatory Markers and Oxidative Stress Mediators

It is well known that inflamm-aging, which is an age-related chronic progressive increase in the pro-inflammatory status, plays an important role in the process of aging and age-related conditions like cognitive decline and chronic comorbidities [8,9]. Several research studies evidenced that healthy aging could be related both to a lower pro-inflammatory status and to an efficient anti-inflammatory response. The imbalance between these two pathways can be a risk factor for frailty and chronic age-related pathologies leading to poor quality of life [10]. Oxidative stress occurs from the imbalance between the production of reactive oxygen and nitrogen species (RONS) and the antioxidant defences. It has been hypothesized that oxidative stress could have a role in the aging process as the age-related modifications could be caused by the accumulation of RONS-induced damages that lead to a progressive loss of function in tissues and organs [11]. Several research studies have investigated the influence of PA on inflammatory and oxidative stress biohumoral markers in the elderly. It has been demonstrated that an active lifestyle can influence the age-related inflammatory profile [12], reducing the secretion of pro-inflammatory factors and increasing the release of anti-inflammatory cytokines. Nevertheless, the effect of different type and intensity of exercise on these pathways has not been fully clarified yet. Monteiro-Junior et al. performed a systematic review and meta-analysis on studies investigating the effect of chronic exercise on interleukin-6 (IL-6), tumor necrosis factor-alpha (TNF-α), and C-reactive Protein (CRP) in a population of ≥60 elderly persons (8 articles were analyzed after screening and application of inclusion/exclusion criteria) [13]. IL-6 and CRP but not TNF-α significantly decreased after the exercise intervention (overall effect *p* < 0.05). A systematic review by Cronin et al. investigated the effect of aerobic and resistance exercise on inflammatory markers (CRP, IL-6, interleukin-8 (IL-8), interleukin-1 beta (IL-1β), and TNF-α) in healthy, physically inactive subjects (11 articles included). Results from studies investigating an elderly population (3 studies of the 11) showed the greatest reduction of inflammatory markers (CRP and IL-6), while results from studies including younger subjects were inconsistent. These different results in the different age groups are probably due to higher basal level of these markers in the elderly and to the resulting higher potential for them to be lowered by PA in this population [14]. Another cross-sectional study investigated the association between inflammatory markers (IL-6 and soluble receptor for TNF-α (sTNFR1)) and muscle/functional performances (assessed by 10 m gait speed) in 221 community-dwelling elderly women aged of ≥65 years. Results did not show any negative correlation between levels of IL-6 and sTNFR1 and muscle or physical performance, probably because the levels of these mediators were not high enough to influence muscles and functionality of the sample [15]. A cross-sectional (1139 subjects included) and longitudinal study (490 subjects with two measures of PA one year apart were included) on elderly men [16] investigated the influence of different levels of PA and sedentary behaviour (SB) on markers of inflammation (IL-6, CRP, and tissue plasminogen activator (tPA), von Willebrand factor (vWF), D-Dimer (ng/mL), and insulin-like growth factor-1 (IGF-1). PA and SB were measured using Actigraph GT3X accelerometers. Results showed that the individuals who spent more time in Moderate to Vigorous PA (MVPA) had lower levels of IL-6, CRP, tPA, vWF, and D-dimer and higher levels of IGF-1 (*p* ≤ 0.006) in contrast with men with higher levels of SB that resulted in having higher levels of IL-6, CRP, tPA, and D-dimer and lower levels of IGF-1 (*p* ≤ 0.03). Moreover, each additional 10 min of MVPA showed to have a lowering effect on IL-6, CRP, tPA, vWF, and D-dimer (3.2%, 5,6%, 2.2%,1.2%, and 1.8% respectively) that, for CRP, vWF, and D-dimer, was independent from levels of SB. A cross-sectional study performed by Do-Yeon et al. [17] investigated the association of quality of diet and physical performance assessed by the Short Physical Performance Battery (SPPB) with IL-6 and TNF-α in 78 frail, elderly South Korean individuals aged ≥ 65 years. The results evidenced that high physical performances (as assessed by SPPB) were associated with lower levels of TNF-α (*p* = 0.001).

Plasma levels of IL-6 and TNF-α were also measured in a population of elderly subjects with hypertension undergoing aerobic training or aerobic and resistance training for 10 weeks. In the group of subjects that underwent both aerobic and resistance training, TNF-α levels were lower (*p* = 0.01), while IL-6 was reduced in subjects undergoing aerobic training compared to untrained controls (*p* = 0.04) [18]. Moreover, direct measurement of the levels of oxidative products has been carried out in some studies. Alghadir et al. showed that, in a population of elderly undergoing a 24-week program of PA, there was a significant decrease in oxidative stress markers, including high sensitivity C-reactive protein (hsCRP), malondialdehyde (MDA), and 8-hydroxyguanine (8-OHdG) when compared to subjects who did not exercise [19]. Similarly, a 12-week program of Nordic walking in elderly women showed a decrease in levels of MDA oxidation products (*p* = 0.01) [20]. In another study, a thorough evaluation of the oxidative status of the included subject (61 women and 34 men aged ≥ 60 years) was carried out through determination of plasma total antioxidant status (TAS), plasma antioxidant enzyme activities, i.e., glutathione peroxidase (GPx), catalase (CAT), superoxide dismutase (SOD), and membrane lipid peroxidation (TBARS). Accelerometers were used to evaluate PA. Among the female sample, TAS was significantly lower and CAT activity was significantly higher in the group that met the criteria of recommended levels of PA for healthy adults (daily step goal of 10,000 steps) than in the group that did not meet these criteria. Correlation analysis showed an inverse association between PA and TAS, while moderate to vigorous physical activity (MVPA) was related to an increase in GPx antioxidant activity in the elderly women sample. In elderly male subjects, a significant correlation was found between CAT activity and the level of PA related to the lifestyle [21]. On the same research line, in a large study involving 1449 subjects, SOD activity and plasma levels of malondialdehyde (MDA) and 4-hydroxynonenal (4-HNE) were determined. Results from a subpopulation of elderly subjects with hypertension showed that different types of PA induced an improvement in antioxidant activity and a reduction in MDA levels; however, these results were not always significant for all the types of PA advocated [22]. Other direct markers of inflammation are those related to the activation of the immune system, including the toll-like receptor (TLR) on white blood cells. This marker has been investigated in a clinical trial, evaluating the effect of an 8-week program of resistance training in elderly subjects. Results showed that, in those patients that completed the program, the expression of TLR-2 and TLR-4 was reduced (*p* < 0.04 and *p* < 0.03, respectively). Furthermore, C-reactive protein levels also decrease in the training group [23]. Similarly, another trial by the same research group demonstrated that whole-body vibration training had a significant effect in the reduction of TLR-2 and TLR-4 in a population of elderly subjects [23]. Furthermore, a cross-sectional study which evaluated TLR activity and cytokines expression reported results stratified by Metabolic Equivalent of Tasks (MET), showing that elderly subjects with higher MET levels and exercise levels had decreased blood levels of IL-6 (*p* = 0.001) but not of TNF-α (*p* = 0.148) and myeloperoxidase (*p* = 0.799). TLR-2 levels significantly decreased both in males and females, according to MET levels, while TLR-4 did not show any significant decrease [24]. Results of the included studies were reported in Table 1.

### 3.2. Effect of PA on Cardiac Biomarkers

A sedentary lifestyle and the lack of a scheduled activity during daily life are well-established risk factors for cardiovascular disease (CVD) because of negative effects on cardiac and endothelial function, including a pro-atherogenic action [26,27]. The classical and nonclassical risk factors for CV disease were investigated in a recent paper, where the single factors were measured at different time-points of a specific PA program in a sample of elderly women (aged 65.0 ± 7.3 years). Body mass index (BMI), waist and hip circumferences, resting systolic and diastolic blood pressure (BP), and resting heart rate were significantly reduced after 2 weeks of a program based on general fitness, yoga, body balance, and self-guided PA. Exercise capacity, low-density lipoprotein (LDL) and high-density lipoprotein (HDL-C), cholesterol, and other atherogenic lipid indices (ALI) also improved after 2 weeks. At three months of the PA program, the values of these markers improved even more, with a significant reduction of calculated CVD risk at ten years [1]. PA also stimulates endothelium to synthesize and release the tissue-plasminogen activator (tPA), and some evidence suggested that also E-selectin may be influenced by PA [26]. A plethora of factors are actually influenced by PA, including C-reactive protein (CRP), interleukin-6 (IL-6), tPA, E-selectin, and adipokines, and several studies attempted to address the influence of PA on the cardiovascular system. A recent paper by Elhakeem et al. evaluated heart rate and data collected by movement sensors to derive overall PA energy expenditure (KJ/kg per day) and time spent in sedentary behaviours (<1.5 metabolic equivalent of tasks), in light PA (1.5–3 metabolic equivalent of tasks), and in moderate-to-vigorous intensity PA (>3 metabolic equivalent of tasks). Results of the linear regressions analysis showed a significant association between time spent in PA (both in light and moderate to vigorous) and blood levels of CRP, IL-6, leptin, and adiponectin, especially in women [26]. Furthermore, the same study reported a positive association between cardiorespiratory fitness and favourable biomarkers levels. Among other biomarkers, a recent cross-sectional study on 1130 men evaluated the influence of PA on the behaviour of N-terminal pro-brain natriuretic peptide (NT-proBNP) and high sensitivity Troponin T (hsTnT), which are both markers of cardiac injury. The results showed that the total amount of PA in patients aged 70–91 years was nonlinearly associated to lower NT-proBNP and hsTnT. Higher levels of PA were associated with lower levels of NT-proBNP but significantly only below a certain threshold of activity per day (measured by accelerometer count, step count, and minutes of moderate/vigorous activity). Similarly, PA level was also associated with lower levels of hsTnT, with significant correlations only below threshold levels of counts, steps, moderate/vigorous activity, and light activity [16]. On the same research line, Van der Linden et al. hypothesized that the frail elderly population is characterized by high levels of basal cardiac Troponin T (cTnT) and may benefit from the potential effects of an exercise intervention. The greatest part of the population evaluated had cTnT levels above the 99th percentile. However, results showed no evident effect of a 24-week resistance-training program on the cTnT levels [28]. Results of the included studies were reported in Table 2.

### 3.3. Effect of PA on Metabolic Parameters

There is a consistent and rising evidence that metabolic pathways and endocrine system as well as immune system and defense mechanisms are definitely affected by the aging process. Specifically, in the elderly, the carbohydrates metabolism is impaired and utilization of blood glucose is decreased [30]; furthermore, in this population, lipid profile results were imbalanced by the altered fat utilization and the lack of PA [31]. Vitamin D levels, the main factor influencing bone health, also result in impairment for a plethora of alterations in kidney and other endocrine organs [32]. There is a growing interest and development of new data analysis methods for the assessment of metabolic status and metabolic changes that occur in the individual during and after activities [33]. It is a common hypothesis that the prevention of metabolic noncommunicable diseases passes through the opportunity to understand which kind of activities may be useful in improving the metabolome of the elderly. In a recent study based on aged Korean woman, the effect of combined aerobic and anaerobic exercise on glucose metabolism was investigated by assessing insulin resistance, Growth Hormone (GH), Insulin-like growth factor-1 (IGF-1), Deidrossiepiandrostenedione (DHEA-S), and estrogen values. The authors found that blood glucose levels decreased significantly when compared to non-exercise controls while GH and DHEA-S increased. Interaction effects were found for IGF-1, GH, and DHEA-S. From these results, it seems that, in older women, the combination of aerobic and anaerobic exercise improves insulin resistance, avoiding the decline of glucose metabolism function [30]. Combined exercise was also investigated in another study; specifically, the effect of resistance training and multicomponent exercise was compared. Main findings included the evaluation of aerobic functionality, lipid profile, and inflammatory markers. The results showed that only epidermal growth factor (EGF) levels and adiponectin (ADN) levels were different between groups, with an increase of the EGF in the group of elderly undergoing multimodal fitness program and a significant ADN reduction in the resistance training group [31]. In a study carried out on 85 healthy older subjects, the levels of vitamin D, creatinine kinase, lactic acid dehydrogenase, troponin I, total antioxidant capacity, body composition, and PA were evaluated to find possible correlations among these parameters. Main findings showed that, in physically active subjects, there was a significant increase in the vitamin D serum levels, calcium, and total antioxidant capacity, with an associated reduction in the levels of muscle fatigue biomarkers: creatine kinase, lactic acid dehydrogenase, troponin I, and hydroxyproline. Based on these results, improved biohumoral markers of bone and muscle health correlated with the improvement in muscle relief and performance of physically active participants [32]. Similarly, a study investigating the beneficial effects of Tai-Chi in older adults showed a statistically significant difference in glycosylated haemoglobin levels compared to untrained controls as well as a significant increase in total antioxidant status [25]. Another study evaluating the association between peak oxygen uptake (a marker of aerobic capacity) and metabolic/cardiovascular parameters showed that subjects with lower VO_2_ had a higher risk to present altered cardiovascular parameters and had higher levels of accumulation of wide-spectrum acyl-carnitines, alanine, and glutamine [29]. The role of microRNA in diabetes mellitus type 2 (T2DM) is growing in interest in the scientific literature. Reduced levels of the microRNAs miR-146a and miR-155 contribute to a pro-inflammatory state associated with T2DM. The effect of strength and cardiovascular training on T2DM patients has been investigated compared to a group of nondiabetic subjects, showing a significant increase of miR-146a and a decrease of blood glucose levels, which were more pronounced in diabetic patients after strength training [34]. In a Swedish study aimed at evaluating the wellbeing of the elderly, the Psychological General Wellbeing (PGWB) index has been put into correlation with several metabolic biomarkers, including the levels of several types of lipoproteins, BMI, and blood pressure. The only significant results found was a positive association between the high-density lipoprotein and the level of general health, according to the PGWB [35]. Caminiti et al. investigated hormonal responses (levels of total and free testosterone, IGF-1, GH, and Sex-Hormone binding protein (SHBG)) after two different types of aerobic PA (interval training (IT) and continuous training (CT)) in chronic heart failure (CHF) elderly patients. Results showed a greater increase in total and free testosterone levels and in IGF-1 in the IT group compared to the CT group. On the contrary, levels of testosterone and IGF-1 remained unchanged after CT. GH significantly increased and SHBG decreased in both groups without between-groups differences. The level of hormonal response was related neither to an improvement of exercise capacity nor to the training load, but it seemed to be related to exercise intensity [36]. Results of the included studies were reported in Table 3.

### 3.4. Effect of PA on Other Biohumoral Markers

A plethora of other biomarkers has been investigated to understand and highlight the effect of PA on their levels. In a cohort of postmenopausal women with breast cancer, the levels of progesterone, estrogens, and their precursors were measured on tissue samples and correlated to BMI and PA status. An interesting result is that estradiol, estrone, and testosterone levels were significantly associated with BMI in women with Estrogen Receptor positive (ER+) breast cancer. Furthermore, an inverse association was found between time spent in PA and serum estradiol levels among ER+ subjects, although this difference was not significant on tissue samples [37]. Another relevant biomarker that has been recently proposed as a predictor of changes in physical function is the C-terminal Agrin Fragment (CAF), a product of the catabolism of neuromuscular junction molecule Agrin. A cohort of 333 older subjects has been followed for 1 year to evaluate the effect of a 12-month program of health education only or a 12-month program of walking, strengthening, flexibility, and balance exercises. However, the large trial failed to demonstrate a significant association between CAF levels and the 12-month activity program [38]. Two large studies investigated the role of PA on kidney function. A study on 1041 older subjects investigated the association between muscular strength and kidney disfunction, by measuring cystatin C levels and maximal muscle strength. The odds ratio of having elevated cystatin C was higher in those subjects with lower muscle strength. Similarly, those subjects with lower muscle strength had also lower estimated glomerular filtration rate from cystatin C (eGFRcysC) [39]. The second study evaluated a cohort of 1352 men, in which the higher levels of PA and lower levels of sedentary behaviours reduced the odds ratio for decreased eGFR [16]. Moreover, a recent paper investigated the possible role of PA on renal proximal tubule stress levels through the evaluation of urinary levels of liver-type fatty acid-binding protein (L-FABP). The intent of the study was two-fold: in a cross-sectional evaluation, urinary L-FABP levels were significantly lower in those subjects with higher PA levels than in those with lower PA levels; in the interventional study, those subjects that underwent 12 weeks of aerobic training had significantly decreased levels of urinary L-FABP [40].

## 4. Discussion

There is growing evidence on the effects of PA in the elderly, and the clinical tools for the assessment of these effects are the focus of several recent research studies. According to the results of the present review, there are many distinct biomarkers influenced by PA in the elderly, although none of them have sufficient evidence for clinical use as the majority of these biomolecules have been investigated in one or very few studies. However, promising results are available, especially concerning the metabolic and cardiovascular indexes, as a growing number of studies demonstrate the role of PA on improving parameters related to heart function and CV risk like atherogenic lipid profile [41]. Prospective observational studies confirm that high–moderate levels of leisure time PA are able to decrease the risk of CV disease in both sexes with an effect size that ranges between 20–30% and 10–20% respectively, showing a dose–effect relationship [41]. Research studies have evidenced that the association between higher levels of PA and lower CV disease rates can be explained in large part by the reduction of known risk factors, with inflammatory/hemostatic biomarkers making the largest contribution to lowered risk, followed by a positive effect on blood pressure, lipids, and body mass index [42]. Furthermore, it is also a verified hypothesis that PA is able to modify the inflammatory status of elderly subjects by decreasing the levels of pro-inflammatory cytokines, including IL-1, IL-6, and TNF-α [13,14]. Several studies have investigated the effect of exercise on inflammatory factors; it has been evidenced that acute bouts of exercise result in a transient, mostly pro-inflammatory effect, which is proportional to the amount of exercise and to the entity of muscle injury [43,44]. On the other hand, regular PA has been associated with a chronic anti-inflammatory effect. The mechanisms underlying this action are not well defined and include reduction of body weight [45], reduction of basal levels of pro-inflammatory cytokines and pro-atherogenic adipokines, enhancement of the expression of antioxidant and anti-inflammatory mediators in the vascular wall, and insulin-sensitizing pathways [46,47]. Moreover, regular exercise has been demonstrated to be able to attenuate the age-associated increase in oxidative stress and nuclear factor-κB activation in animals [48] and to reduce toll-like receptor signaling [23], which may explain its chronic anti-inflammatory effect. 

As for the influence of PA on oxidative stress and antioxidant systems, a recent review by Nocella et al. [7] highlighted that high-intensity physical exercise can cause redox imbalance, leading to several types of injuries and muscle damage, while the studies reported in this review show an improvement in antioxidant activity and reduction of oxidative stress in elderly performing regular PA. Similar to our results, some studies demonstrated that exercise and regular PA have a positive impact on oxidative stress and inflammation during aging [49,50]. The balance between Reactive oxygen species (ROS) production and antioxidant systems is a very important, as ROS plays a dual role: at low or moderate levels, they have a beneficial action on cellular responses, while at high concentrations, they cause inflammation and oxidative damage to cells and tissues [51,52]. This is crucial for the aging process, as inflammatory processes and oxidative stress are biochemical alterations related to the etiology and complications of several age-related diseases such as T2DM, Alzheimer’s disease, CV disease, and cancer. It has been evidenced that regular practice of PA has a positive effect on the aging process, with an action on biochemical changes related to aging and on the risk of chronic diseases. This action has the consequence of enhancing quality of life in aging individuals and of increasing longevity [53,54]. It has also been evidenced that PA could modify blood glucose levels and glycosylated haemoglobin levels in nondiabetic elderly subjects [42]. One study has also demonstrated a significant increase in miR-146a (reduced concentrations of miR-146a contribute to a pro-inflammatory state associated with T2DM) with a decrease in serum blood glucose levels, which was more evident in the group of diabetic patients compared to healthy individuals after strength training [34]. In agreement with our results, literature shows that PA (both endurance and strength training) has an important role in the prevention and control of T2DM, producing acute and chronic physiological effects [55,56]. It has been demonstrated that insulin action in muscle and liver can be modified by acute bouts of exercise and by regular PA. Regular training increases muscle capillary density and insulin signaling proteins [57]. Moreover, it has been evidenced that both aerobic and resistance training promote adaptations in skeletal muscle, adipose tissue, and liver associated with enhanced insulin action, regardless of weight loss [58].

Some studies reported in this review showed that PA was able to raise testosterone, GH, and vitamin D levels in elderly populations [32]. Our results comply with the results of some cross-sectional studies performed on middle-aged and older men which indicate that circulating testosterone concentrations may be higher in men who regularly exercise [59,60], and this is very important in the elderly in order to counteract the physiological age-associated decline of serum testosterone. It should be noted that low levels of testosterone in men have been associated with decreased sexual function, loss of muscle mass and strength, osteoporosis, declining cognitive function, and poorer quality of life [61]. As for the effects of PA on GH levels, neuroendocrine mechanisms underlying exercise-facilitated GH secretion are complex; they probably include somatostatin withdrawal, GH-releasing hormone (GHRH) release, and possibly co-secretagogue actions. The effect of PA on vitamin D levels could be related to the evidence that shows that long-term regular exercise programs are able to increase bone mineral density (BMD) in the elderly and to consequently reduce the risk of osteoporotic fractures [62], which are the most fearsome events in an aged person that frequently lead to disability, hospitalization, and death.

There is also some evidence from literature about the role of PA on other interesting markers like CAF and urinary L-FABP [40], but further studies should be designed to confirm and fully explain these results. 

Concerning the kind of exercise that could have a positive effect on health indicators in the elderly, there is not strong evidence, as the majority of the studies in literature have focused on global levels of PA. Only a few of them investigated the effect of aerobic, resistance or combined training, or specific kind of activities (Tai-chi, Nordic walking, and whole-body vibrations) on biohumoral indicators. Well-designed clinical trials on the effect of a different kind of PA on health indicators are therefore needed to better understand the role of each kind of exercise on specific physiopathological pathways.

Moreover, most of the reported studies investigate a population of “healthy” community-dwelling elderly and only a few of them include people with chronic pathologies (Table 1, Table 2 and Table 3). This scarce evidence on elderly with chronic comorbidities limits the transposition of the results to clinical settings in which the majority of the individuals have multimorbidity [63].

## 5. Conclusions

The studies that are reported in this review give strong evidence of an effect of PA on the health of elderly people that is not generic and confused but could be evidenced and “quantified” by the modifications of the levels of biohumoral indicators. This represents an additional support to the concept of the role of PA in primary and secondary prevention of noncommunicable diseases. Therefore, the practice of PA and the reduction of sedentary behaviors should be encouraged in all ages, overcoming psychological barriers and false beliefs of the elderly. The evidence coming from further clinical research and new biotechnologies could help in having the opportunity to “tailor” the right type of exercise for the particular clinical and genetic features of each individual in order to build up an individualized preventive strategy.

## Figures and Tables

**Table 1:** Inflammatory markers and oxidative stress mediators.

Study	Physical Activity	Participants	Biomarkers	Main Findings
Alghadir et al. 2016 [19]	Moderate aerobic for 24 weeks	100 (age 65–95 y)	MDA, 8-OHdG, TAC, and hs-CRP	Physically active persons showed a higher cognitive performance along with reduction in the levels of MDA, 8-OHdG, and hs-CRP and increase in TAC activity compared with sedentary participants.
Felicio et al. 2014 [15]	Muscle performance and handgrip were measured using dynamometer	221 women (mean age 71 y)	IL-6, SNTFR	IL-6 (0.87 pg/mL) correlated with the power of the knee extensors (*r* = 0.14; *p* = 0.03) and the power of the knee flexors (*r* = 0.16; *p* = 0.01). IL-6, level of physical activity, and depressive status explained 5.5% (*R^2^* = 0.055, *p* < 0.01) of average power of knee extensors variability.
Ferrer et al. 2018 [24]	Metabolic equivalent of task measurement; Minnesota leisure-time physical activity level	116 (age 55–80 y)	IL-6 and TLR protein array	Exercise induced a decrease in the IL-6 circulating levels and the TLR2 protein levels in PBMCs. Anti-inflammatory IL-10 was increased in active subjects.
Fraile-Bermudez et al. 2015 [21]	Level of physical activity measured through accelerometers	61 women and 34 men (mean age 70 y)	GPx, SOD, CAT, and TBARS	In active women, lower levels of TAS were found. Moderate to vigorous physical activity was negatively correlated with TAS but was correlated with increase in the GPx activity. The counts per minute were positively correlated with CAT activity.
Kim et al. 2017 [17]	Grip strength and SPPB	78 (mean age 78.3 y)	IL-6 and TNF-α	Higher SPPB score was associated with lower levels of TNF-α.
Kortas et al. 2017 [20]	Nordic walking for 12 weeks	35 women (mean age 68 y)	MDA and AOP	Statistically significant decreasing of MDA level and concentration of AOP
Lima et al. 2015 [18]	Aerobic training vs. arobic + resistance training for 10 weeks	44 (age 60–75 y	IL-6 and TNF-α	IL-6 was reduced in aerobic training group compared to controls (*p* = 0.04), and TNF-α levels were lower in aerobic + resistance group compared to controls (*p* = 0.01).
Mendoza-Nunez et al. 2018 [25]	Tai-Chi	110 affected by MetS	TAS, TNF-α, IL-6, IL-8, and IL-10	Activity group showed a statistically significant increase in TAS and a decrease in the oxidative stress score (*p* < 0.05).
Parsons et al. 2017 [16]	Level of physical activity measured through GT3X accelerometers	1139 (mean age 79 y)	IL-6, CRP, tPA, vWF, and D-Dimer	Higher physical activity was associated with lower levels of IL-6, CRP, tPA, vWF, and D-Dimer. Furthermore, each additional 10 min of moderate to vigorous activity was associated with a 3.2% lower IL-6, 5.6% lower CRP, 2.2% lower tPA, 1.2% lower vWF, and 1.8% lower D-dimer.
Rodriguez-Miguelez et al. 2014 [23]	Resistance exercise training	26 (mean age 69.5 y)	IL-10, TNF-α, and CRP	TNF-α remained unchanged in both trained subjects and controls. IL-10 was upregulated in trained subjects. CRP values decreased in trained subjects only.
Yu et al. 2018 [22]	Walking, square dancing, Taiji, and yoga	1449 (age 45–79 y), with or without hypertension	SOD, MDA, and 4-HNE	In individuals with hypertension, MDA levels decreased (if walking/square dancing), SOD activity increased (if walking/square dancing), and 4-HNE levels decreased (if Taiji/yoga). In individuals without cardiovascular disease, MDA levels decreased (if any activity), SOD activity increased (if walking/square dancing), and 4-HNE levels decreased (if Taiji/yoga)

Where not specified, the patients where healthy community-dwelling elderly subjects. MDA = malondialdehyde, 8-OHdG = 8-hydroxyguanine, TAS = Total Antioxidant Status, TAC = Total Antioxidant Capacity, and hs-CRP = high-sensitivity C-reactive Protein, MetS = Metabolic Syndrome, tPA = tissue plasminogen activator, vWF = von Willebrand factor, SNTFR = soluble receptor for tumor necrosis factor alpha, AOP = advanced oxidation products, SPPB = Short Physical Performance Battery, GPx = glutathione peroxidase, CAT = catalase and SOD = superoxide dismutase, TBARS = membrane lipid peroxidation, PBMC = peripheral mononuclear blood. Cells, 4-HNE = hydroxynonenal, y = years, TLR = toll like receptor, IL = interleukin-1, TNF-α = tumor necrosis factor-alpha, CRP = C-reactive Protein.

**Table 2:** Cardiovascular risk biomarkers.

Study	Type of Exercise	Participants	Biomarkers	Main Findings
Elhakeem et al. 2018 [26]	Light and moderate-to-vigorous activity, monitored with sensors worn for 5 consecutive days	795 men and 827 women (age 60 to 64 y)	E-selectine, leptin, and adiponectine	Greater time in light PA and moderate-to-vigorous intensity PA and less sedentary time were associated with more favorable biomarker levels.
Koh et al. 2018 [29]	Aerobic capacity (VO_2_), physical activity frequency, intensity, and duration	141 (mean age 70.6 y)	Ecographic and cardiac magnetic resonance imaging parameters	Compared to participants with high VO_2_, participants with low VO_2_ had lower ratio of peak velocity flow in early diastole to peak velocity flow in late diastole by atrial contraction of >0.8 (*p* = 0.001) and lower left atrial conduit strain (*p* = 0.045)
Parsons et al. 2018 [16]	Level of physical activity measured through GT3X accelerometers	1130 men (age 70 to 91 y)	NT-proBNP and hsTnT	For each additional 10 min of moderate/vigorous activity, NT-proBNP was lower by 35.7% and hsTnT was lower by 8.4%, in men who undertook <25 or 50 min of moderate/vigorous activity per day, respectively.
Van der Linden et al. 2014 [28]	24-week supervised resistance-type exercise training program vs. normal activity monitoring	52 pre-frail elderly (age ≥ 65 y)	cTnT	The majority of participants had cTnT levels above the 99th percentile. These data confirm the hypothesis that chronically elevated cTnT concentrations are highly prevalent among (pre)frail elderly subjects.
Zmijewski et al. 2015 [27]	Organized, group-based physical activity	35 women (mean age 65 y)	BP, resting HR, EC, HDL, and LDL	Two-week effects included significant decreases in BMI, waist and hip circumferences, resting BP, and resting HR; improved EC; and improved LDL, HDL, and TC, with a reduction in 10-year estimated risk of death from CVD. Three-month effects included a further decrease in systolic BP, improvements in EC and HDL, and maintenance of lower levels of CVD risk.

Where not specified, the patients where healthy community-dwelling elderly subjects. PA = physical activity, TC = Total Cholesterol, TAG = triacylglycerols, HDL = High-Density Lipoproteins, LDL = Low-Density Lipoproteins, BP = blood pressure, HR = heart rate, EC= exercise capacity, BMI = Body Mass Index, CVD = Cardiovascular Disease, NT-proBNP = N-terminal pro-brain natriuretic peptide, hsTnT = high sensitivity Troponin T, cTnT = cardiac Troponin T.

**Table 3:** Metabolic biomarkers.

Study	Type of Exercise	Participants	Biomarkers	Main Findings
Al-Eisa et al. 2016 [32]	Physical activity assessed through estimated energy expenditure scores	85 (age 64 to 96 y)	TAG, TC, LDL, HDL, 25(OH)D, TAC, CK, LDH, Troponin I, and hydroxyproline	Significant reduction of TC, TAG, LDL, and HDL occurred in subjects with moderately active and active subjects. Significant increase in 25(OH)D and TAC and a reduction in the levels of muscle fatigue biomarkers occurred in physically active subjects.
Biddle et al. 2018 [33]	Physical behaviors (time spent per day): stepping, sleeping, sitting, and standing	435 (mean age 66.7 y)	Fasting and 2 h glucose and insulin levels, and HbA1c	Reallocating 30 min from sleep, sitting, or standing to stepping was associated with 5–6 fold lower 2-h glucose, 15–17 fold lower 2-h insulin, and higher insulin sensitivity.
Ha and Son 2018 [30]	Aerobic + anaerobic exercise for 12 weeks vs. controls	20 Korean women	Insulin resistance, GH, IGF-1, DHEA-S, and estrogen	GH level increased significantly in the exercise group. The DHEA-S level significantly increased in the exercise group. The estrogen level increased significantly in the exercise group.
Hurtig-Wennlof et al. 2014 [35]	International Physical Activity Questionnaire modified for the elderly.	389 community- dwelling elderly (mean age 74 y)	LDL, HDL, Apolipoprotein A1, and B. PGWB	PGWB correlated significantly with all parameters, positively with LDL, HDL ApoA1 (respective Spearman’s rho 0.03, 0.05, and 0.013), and negatively with ApoB (rho −0.031).
Kortas et al. 2017 [20]	Nordic walking for 12 weeks	35 women (mean age 68 y)	TC, TAG, HDL, LDL, and ferritin	The training induced a rise of HDL cholesterol (*p* < 0.05), whereas other lipid parameters remained unchanged. Decrease of blood ferritin (*p* < 0.05)
Leite et al. 2015 [31]	Resistance training vs. multicomponent exercise for 12 weeks	24 women and 15 men (age 65 to 75 y)	LDL, HDL, glucose, TAG, NEFA, adiponectine, ferritin, and EGF	Among the evaluated biomarkers, only high molecular weight adiponectin decreased significantly within the RT group (*p* = 0.03) after the exercise protocol. Between-group differences included only ferritin (*p* = 0.02) and EGF (*p* = 0.01).
Mendoza-Nunez et al. 2018 [25]	Tai-Chi	110 affected by MetS	HbA1c	Decrease in HbA1c concentration was observed in the TC group compared with the control group (*p* < 0.05).
Santos Morais et al. 2017 [34]	Strength training and guided walk (monitored through Polar Team software)	23 (mean age 68.2 y), of whom 13 were diabetics	miR-126, miR-146a, and miR-155 in	Diabetic patients had higher reduction in blood glucose than nondiabetics, which was paralleled by a positive change of the circulating levels of miR-146a but not of the other miRs.

Where not specified, the patients where healthy community-dwelling elderly subjects. MetS = Metabolic Syndrome, TC = Total Cholesterol, TAG = triacylglycerols, HDL = High-Density Lipoproteins, LDL = Low-Density Lipoproteins, HbA1c = glycosylated haemoglobin, GH = growth hormone, IGF-1 = Insulin-like Growth Factor 1, DHEA-S = deidroepiandrosterone sulphate, NEFA = Non Esterified Fatty Acid, EGF = Epidermal Growth Factor, 25(OH)D = 25-hydroxy vitamin D, TAC = Total Antioxidant Capacity, CK = Creatinine Kinase, LDH = Lactic acid DeHidrogenase, VO_2_ = peak Oxigen Uptake, PGWB = Psychological Geneal Wellbeing, miR = micro RNA.
